# Evaluation of the Interplay between the ADAR Editome and Immunotherapy in Melanoma

**DOI:** 10.3390/ncrna7010005

**Published:** 2021-01-12

**Authors:** Marina Tusup, Phil F. Cheng, Ernesto Picardi, Austeja Raziunaite, Reinhard Dummer, Mitchell P. Levesque, Lars E. French, Emmanuella Guenova, Thomas M. Kundig, Steve Pascolo

**Affiliations:** 1 Department of Dermatology, University Hospital of Zürich, Gloriastrasse 31, 8091 Zürich, Switzerland; marina.tusup@usz.ch (M.T.); Phil.Cheng@usz.ch (P.F.C.); austeja.raziunaite@gmail.com (A.R.); reinhard.dummer@usz.ch (R.D.); Mitchell.Levesque@usz.ch (M.P.L.); Lars.French@med.uni-muenchen.de (L.E.F.); Emmanuella.Guenova@usz.ch (E.G.); Thomas.Kuendig@usz.ch (T.M.K.); 2 Faculty of Medicine, University of Zürich, 8091 Zürich, Switzerland; 3 Department of Biosciences, Biotechnology and Biopharmaceutics, University of Bari “A. Moro”, 70121 Bari, Italy; ernesto.picardi@uniba.it; 4 Institute of Biomembranes, Bioenergetics and Molecular Biotechnologies (IBIOM), National Research Council, 70126 Bari, Italy; 5 Department of Dermatology and Allergy, University Hospital, LMU Munich, 80336 Munich, Germany; 6 Department of Dermatology, Lausanne University Hospital and Faculty of Biology and Medicine, University of Lausanne, 1015 Lausanne, Switzerland

**Keywords:** editing, melanoma, ADAR, Alu sequences, immune checkpoint inhibitors, immunotherapy

## Abstract

Background: RNA editing is a highly conserved posttranscriptional mechanism that contributes to transcriptome diversity. In mammals, it includes nucleobase deaminations that convert cytidine (C) into uridine (U) and adenosine (A) into inosine (I). Evidence from cancer studies indicates that RNA-editing enzymes promote certain mechanisms of tumorigenesis. On the other hand, recoding editing in mRNA can generate mutations in proteins that can participate in the Major Histocompatibility Complex (MHC) ligandome and can therefore be recognized by the adaptive immune system. Anti-cancer treatment based on the administration of immune checkpoint inhibitors enhance these natural anti-cancer immune responses. Results: Based on RNA-Seq datasets, we evaluated the editome of melanoma cell lines generated from patients pre- and post-immunotherapy with immune checkpoint inhibitors. Our results reveal a differential editing in Arthrobacter luteus (Alu) sequences between samples pre-therapy and relapses during therapy with immune checkpoint inhibitors. Conclusion: These data pave the way towards the development of new diagnostics and therapies targeted to editing that could help in preventing relapses during immunotherapies.

## 1. Introduction

The diversification of messenger RNA (mRNA) sequences from genomic DNA relies on posttranscriptional mechanisms, such as alternative splicing and RNA editing [1,2]. In mammals, RNA editing involves the deamination of adenosines (A) or cytidines (C) generating in situ inosine (I) or uridine (U), respectively. While adenosine deaminase acting on RNA (ADAR) [3] and adenosine deaminase acting on tRNA (ADAT) [4] deaminate A residues, enzymes from the APOBEC family [5] deaminate C residues. In the following, we focus on ADAR activities, as A-to-I modifications globally represent 97% of RNA editing events [6], in mammals and especially in humans. A-to-I editing events can occur in coding and non-coding sequences of mRNA [7]. In general, they are prominent in double-stranded RNA stretches formed by inverted non-coding repeats such as Arthrobacter luteus (Alu) and long interspersed element (LINE) located in mRNA untranslated regions (UTRs) and introns [8,9,10]. RNA editing in non-coding sequences can influence alternative splicing, nuclear retention, and transcript degradation (e.g., recognition by miRNA) [11]. In addition, RNA editing can affect sites in the protein coding region of mRNAs, leading to potential amino acid changes, known as recoding editing [12]. In humans, recoding editing is rare while A-to-I in Alu elements is abundant and accounts for 97% of all available events [13]. Both ADAR1 and ADAR2 can perform recoding or non-recoding editing. A third ADAR protein called ADAR3 is expressed only in the brain and its deaminase activity has not been yet proven [7].

A-to-I RNA editing is essential in maintaining cellular homeostasis [14,15,16] and has been implicated in several diseases ([17] reviewed in [18,19,20]). In particular, the disruption of the controlled expression of ADAR1 and ADAR2 has been shown to contribute to cancer pathogenesis. Based on current reports, recoding and non-coding editomes with hypo- or hyper-editing levels appear to be dependent on cancer-types and genes [21,22]. For example, the DNA base excision repair glycosylase enzyme NEI-like protein 1 (NEIL1), encoding antizyme inhibitor 1 (AZIN1), Ras homologue family member Q (RHOQ), and protein tyrosine phosphatase non-receptor type 6 (PTPN6) are found hyper-edited in cancer compared to healthy tissues, while gamma-aminobutyric acid type A receptor alpha 3 subunit (Gabra3), glutamate receptor subunit B Glur-B (also known as GRIA2), and insulin-like growth factor-binding protein 7 (IGFBP7) are found to be hypo-edited in cancer compared to normal tissue. These editing events have been reported to impact protein functions. Other recoding editing, such as filamin B (FLNB), cyclin I (CCNI), coatomer protein complex subunit α (COPA), and the component of oligomeric Golgi complex 3 (COG3) have not been functionally characterized. Non-coding editomes in cancer can also be different when compared to the corresponding healthy tissues, such as with hypo-editing for melanoma and glioma or hyper-editing (of microRNA) in non-small cell lung carcinoma (NSCLC). This editome is involved in cancer development, progression, invasion, metastatic potential, recurrence, and resistance (reviewed by [18]).

ADARs are also associated with immunity in several ways. First, the elongated form of ADAR1 is located in the cytosol and is induced by type I interferon, while constitutive ADAR2 and the short form of ADAR1 are nuclear. Second, by editing dsRNA, ADARs avoid the stimulation of innate immune responses by endogenous dsRNA [23] and thereby, lowering ADAR1 increases tumor inflammation [24,25]. Third, adaptive immunity is impacted by ADARs; a recent report shows that peptides containing amino acids generated through ADAR-recoding events are human leukocyte antigen (HLA) ligands. In particular, CCNI-edited peptides act as cancer antigens capable of activating tumor infiltrating lymphocytes (TILs) and thereby mediate cancer cell death in melanoma [26]. Thus, ADAR-recoding can impact the Major Histocompatibility Complex (MHC) ligandome and thereby the specific anti-cancer T-cell response.

Although ADARs affect immunity, studies investigating the potential relationship between editome and cancer immune evasion are lacking. Melanoma is the most aggressive form of skin cancer and is currently best treated through the administration of immune checkpoint inhibitors: monoclonal antibodies blocking negative signals generated by cytotoxic T-lymphocyte-associated protein 4 (Ipilimumab) or programmed cell death-1 (e.g., Pembrolizumab or Nivolumab) in activated T lymphocytes [27]. Since such therapies rely on the potential for the immune system to fight cancer cells, and since ADARs are involved in the immune response in multiple ways (see above), we used RNA-Seq datasets to analyze the editome of melanoma cell lines made from tumors obtained before immunotherapy and from Ipilimumab and Pembrolizumab-resistant (relapsing) metastasis.

## 2. Materials and Methods

### 2.1. Patients and Cell Lines

The study was approved by the Kantonale Ethikkommission under approval number KEK-ZH Nr. 2014-0425, EK647 and EK800. After informed consent was given by the patients, biopsies were used to generate melanoma cell lines as described [28]. DNA was used to prepare a customized target library using the Nimblegen SeqCap EZ kit (Nimblegen). Sequencing was performed on an Illumina Hiseq 4000 machine at the Functional Genomics Center of the University of Zürich (FGCZ). For targeted sequencing, we sequenced 0.125 lanes per sample, paired-end, with 150 bp reads [28].

Cell line purity was estimated based on the mutant allele frequency (MAF) calculated as follows: MAF = mutant copies/(wild-type copies + mutant copies). Mutations in BRAF (V600E) and NRAS (Q61R, Q61K, Q61L) in melanoma are reported to be heterozygous, and thus cell line purity was calculated as 2 × MAF. RNA was extracted with the QIAGEN RNeasy kit. RNA capture was performed with the TruSeq RNA library Prep Kit v2 (Illumina). RNA sequencing was sequenced at 125 bp paired end on a HiSeq4000 at the FGCZ.

Datasets for 14 patients were available for analysis. The samples were grouped by treatment: 12 samples were assigned to the group not yet subjected to Ipilimumab and Pembrolizumab therapy (12 cell lines taken from 10 patients) and 11 samples were assigned to the relapsed group (11 cell lines taken from 4 relapsing patients during treatment by Ipilimumab and Pembrolizumab). Those relapses happened during immunotherapy as they appeared within less than one month after the last antibody injection. For two patients, we collected cell lines before immunotherapy and at the point of relapse.

Raw sequencing reads and RNA counts can be retrieved with GEO accession number GSE162095 (https://www.ncbi.nlm.nih.gov/geo/query/acc.cgi?acc=GSE162095).

### 2.2. Bioinformatics and Statistics

Gene-level counts were quantified from pair-end reads aligned to the GRCh38 genome using the quantMode option implemented in STAR (it enables the aligner to count number reads per gene while mapping) [29]. A differential gene expression analysis was performed using Qlucore software v 3.5. and using the R script for limma-voom [30]. Significant differential expression was set to *p* < 0.05 and the false discovery rate *q* < 0.05. Gene expression levels for the ADAR genes were obtained from our USZ melanoma website http://phil.shinyapps.io/ZMCE. Differences between the two groups of patient samples were analyzed through an unpaired *t*-test conducted in GraphPad where *p* < 0.05 values were considered significant. RNA editing events were quantified with ExpEdit [31,32,33,34], a web interface based on REDItools [31]. The program explores known human RNA editing positions annotated in DARNED (approximately 333,215 edited sites, of which 221,595 are A-to-G edited sites) from FASTQ RNA-seq input files. The default settings displayed by the interface were selected for analysis: the qPhred score was set to ≥10 (base quality score), and the mapping quality score was set to ≥20 [26]. The obtained files were adapted for further analysis with Qlucore software v 3.5 focusing on editing events of ≥10%. Recoding and non-recoding editing was analyzed separately with the exclusion of known Single Nucleotide Polymorphism (SNPs) from dbSNP and of any nucleotide changes that did not resemble A to I (G) [9]. Principal component analysis (PCA) plots and heat maps applying hierarchical clustering were generated using Qlucore software. The Alu editing events were analyzed with an unpaired *t*-test with the Benjamini and Hochberg false discovery rate multiple testing correction where significantly different Alu editing was measured with a *p* value of <0.05 and a *q* value of <0.1. Alu editing indexes were calculated as the ratio of the sum of the reads with edited sites (I = G in DARNED) to the total number of reads [22]. Correlation plots were analyzed in GraphPad applying Pearson correlation, where a *p* value of <0.05 was considered significant.

## 3. Results

We analyzed 23 RNA seq; 12 datasets were collected from cell lines made from biopsies before the administration of immune checkpoint inhibitors (“before IT”), and 11 datasets were collected from cell lines made from biopsies of relapsing melanoma in patients treated with Ipilimumab and Pembrolizumab (“relapsed”) (Table 1). Clinical features of the melanoma patients from which cell lines were derived are indicated for each patient in Table 1 and for both groups in Table 2. Our methods to establish cell lines were optimized so that melanoma cells were more effectively retrieved and that the cell culture represented the full range of in vivo tumor heterogeneity [28]. It is not possible to verify that the cell lines will have similar gene expressions or editing profiles than tumor cells in the biopsies, however as all cell lines analyzed here were produced in the exact same conditions, we would expect that if the cell culture derivation would affect gene expression or editing, it would be similar for all, thus not affecting the comparison between the two groups. From a multivariant analysis of limma and voom, no gene was found to be significantly up- or downregulated in one group or the other when *p* values of less than 0.05 and q values of less than 0.05 were used (Supplementary Figure S1A and Supplementary Table S1). In addition, no clear clustering of the two groups can be evidenced using PCA (Supplementary Figure S1B). Thus, we extended our transcriptome analysis by exploring the role of ADARs in checkpoint inhibitor immunotherapies against melanoma (Supplementary Table S2).

### 3.1. Expression of the ADAR Genes before IT Versus in Relapsing Tumours

**Figure 1:**
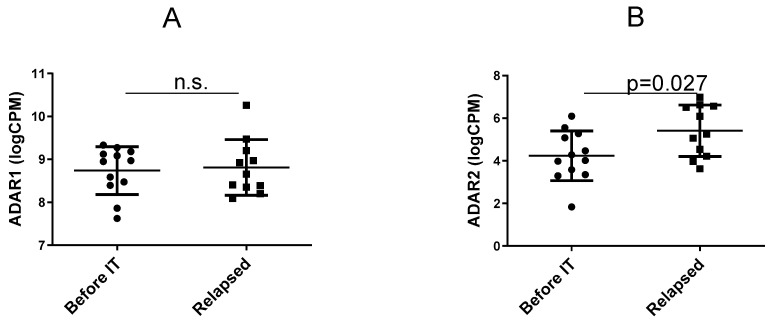
Adenosine deaminase acting on RNA (ADAR) gene expression. The graphs show for each cell line the gene expression of ADAR1 (in **A**) and ADAR2 (in **B**) in the two groups: before immunotherapy (“Before IT”) and in relapse during immunotherapy (“Relapsed”). The *p* value calculated from the unpaired *t*-test is indicated (“n.s.” stands for non-significant).

We evaluated changes in gene expression between the two groups for the *Adar1* and *Adarb1* genes corresponding to the ADAR1 and ADAR2 enzymes, respectively. The analysis shows that ADAR1 gene expression (long and short forms of ADAR1 combined) remained unchanged between the groups (Figure 1A), while ADAR2 was significantly enhanced in the “relapsed” group (Figure 1B) based on an unpaired *t*-test. This result was unexpected, as ADAR2 was previously reported to act as a tumor suppressor [35,36] while ADAR1 has been preferably deemed a tumor oncogene (reviewed by [18]). Thus, the role of ADARs in relapse during therapy by immune checkpoint inhibitors may not be the same as the role of such enzymes in the natural course of melanoma development.

### 3.2. Recoding Editing

**Figure 2:**
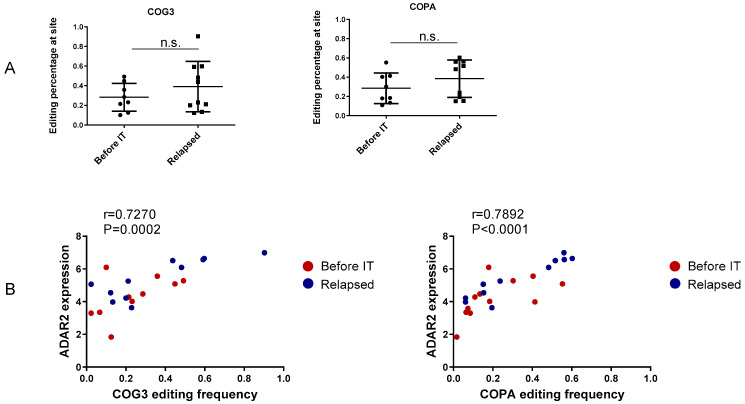
Frequency of recoding editing in component of oligomeric Golgi complex 3 (COG3) and coatomer protein complex subunit α (COPA). The graphs shown in (**A**) report the percentage of recoding editing observed in COG3 (position 46090371) and COPA (position 160302244) for the two groups: before immunotherapy (“Before IT”) and relapse during immunotherapy (“Relapsed”). The correlation plots given in (**B**) depict the level of ADAR2 expression relative to the editing frequencies of COG3 and COPA. Correlation coefficient “r” and Pearson correlation value “*p*” value are indicated. Each dot shown in the figures corresponds to one cell line. n.s.—not significant.

Clinically relevant recoding editing in cancer has been reported. Corresponding levels of editing are shown in Supplementary Figure S2 and Supplementary Table S3. ADAR2 is responsible for most recoding editing events including COG3_I635V and COPA_I164V [37]. While ADAR2 is upregulated in “relapsed”, the two forms of recoding editing were not significantly different between “before IT” and “relapsed” (Figure 2A). These forms of recoding editing show a strong positive correlation with the expression of ADAR2, confirming their ADAR2 dependence (Figure 2B). The recoding editing of COG3 and COPA was first identified as clinically relevant in more than one form of cancer in Han et al. [21]. The editing of COG3 and COPA has been shown to correlate with shortened progression-free survival in renal clear cell carcinoma, and high levels of COG3 editing have been associated with resistance to fluorouracil and austocystin D while high levels of COPA editing have been significantly associated with resistance to austocystin D and lapatinib [37]. Meanwhile, it has been reported that RNA editing at both CCNI R75G and COPA I164V generates MHC-associated epitopes [26]. Our data suggest that in patients treated with immune checkpoint inhibitors, MHC epitopes derived from edited COPA are not strongly immunogenic, as otherwise they would be downregulated when the immune response is enhanced by blockade of immune checkpoint inhibitors.

### 3.3. Non-Recoding Editing in Alu Elements

**Figure 3:**
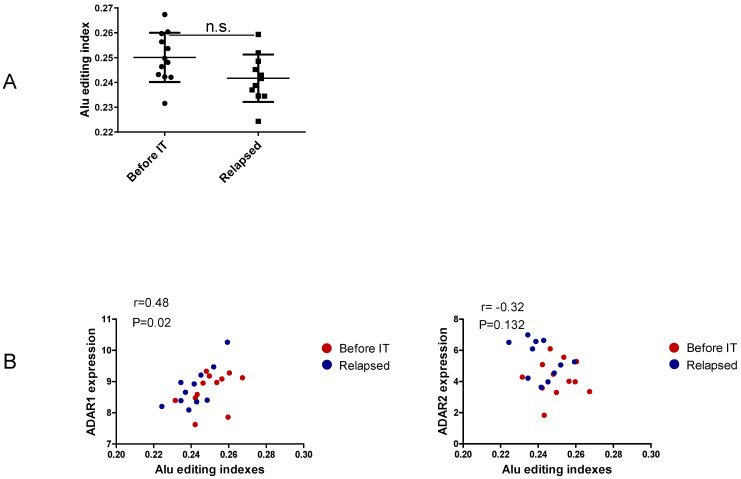

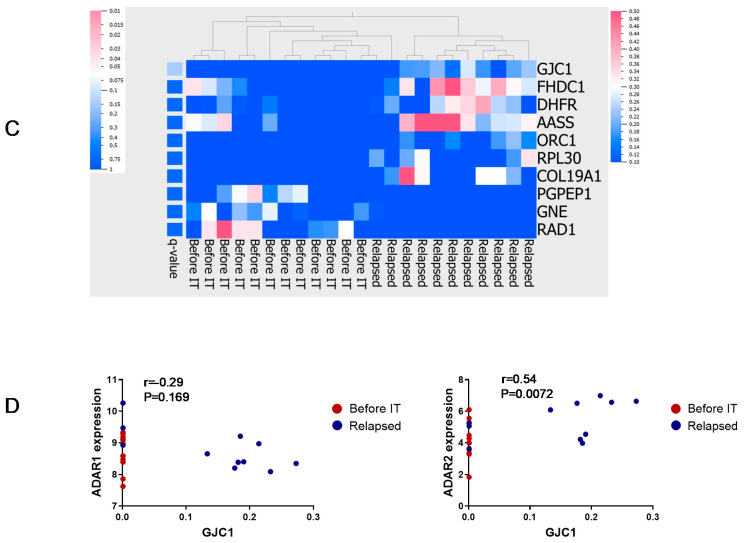
Non-coding editing in Alu elements. Alu editing indexes were calculated for each cell line collected from tumors before immunotherapy (“Before IT”) or from tumors relapsing during immunotherapy (“Relapsed”). The graph shown in (**A**) represents the Alu editing index for each cell line of each group (non-significant *p* value according to an unpaired *t*-test). The correlation plots given in (**B**) report the Alu editing indexes according to ADAR1 or ADAR2 expression. The heatmap given in (**C**) indicates the level of Alu editing observed in genes with a qPhred score of ≥10. The graphs shown in (**D**) report the Alu editing frequency observed in Gap junction gamma-1 protein (GJC1) compared to the level of ADAR1 and ADAR2 expression. In the correlation plots, correlation coefficient “r” and Pearson correlation value “*p*” are shown. Each dot in the graphs or plots corresponds to one cell line. n.s.—not significant.

The global noncoding editome represented by the Alu editing indexes shows no differences between the two groups (Figure 3A and Supplementary Table S4). Additionally, in line with previous reports, we find a positive correlation between ADAR1 expression and Alu editing indexes (Figure 3B), confirming ADAR1’s major contribution to the noncoding editome. However, a focus on non-recoding single editing sites based on a qPhred score of ≥10 indicates Alu hyper-editing in relapsed samples of Gap junction gamma-1 protein (GJC1) at site 42877641 (Figure 3C and Supplementary Tables S5 and S6). Interestingly, GJC1 Alu editing frequency at this site shows no correlation with ADAR1 expression, but it correlates positively with ADAR2 expression (Figure 3D). This editing of Alu elements has been observed for nuclear-retained Cat2 transcribed nuclear RNA [38]. Furthermore, Alu editing in GJC1 is reported as clinically relevant, showing differential editing levels across tumor subtypes and tumor stages and correlating with patients’ overall survival rates [21]. However, further studies must be conducted to determine the contributions of this Alu editing to cancer progression and to responses to therapy.

### 3.4. Principal Component Analysis

**Figure 4:**
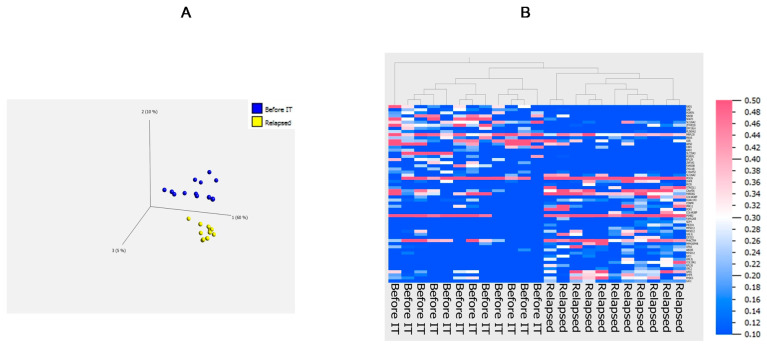
Treatment-based distribution for Alu editing events. A 3D principal component analysis (PCA) plot representation of samples is presented in (**A**). Cell lines are made from tumors before immunotherapy (“Before IT”) or from tumors relapsing during immunotherapy (“Relapsed”). A clear separation of samples based on 57 different Alu editing sites is shown. For the 57 Alu editing sites hierarchically clustered, a heatmap representation is given in (**B**). The scale on the right denotes the editing frequency.

To obtain an overview of potential differences in editing patterns observed between “Before IT” tumors and “Relapsed” during treatment with checkpoint inhibitors, we applied a principal component analysis. It was done using the two group comparison statistical analysis (*t*-test) provided by the Qlucore software and the following parameters: filtering by variance 0.15, *p* value was 0.05, and q value was 0.775. The outcome by the software was 57 Alu editing sites in 57 genes for the sample representation in a 3D PCA plot and heatmap representation of Alu editing sites (Figure 4 and Supplementary Table S7). Editing events were not normalized as a single editing site was used as a unique identifier for variable analysis and not the complete gene with all the editing events. From the PCA plot including the 57 Alu editing events, we can clearly separate the two groups of patients based on the corresponding cell lines. This separation is confirmed in a hierarchically clustered heatmap showing that Alu signatures can differentiate cell lines from patients who relapse from cell lines taken from patients before IT (Figure 4A,B). Conversely, no recoding editing signatures could be identified to separate the two groups (Supplementary Figure S2).

Collectively, our study reveals an editing signature in Alu elements that characterizes tumors relapsing during treatment with immune checkpoint inhibitors (Figure 3C and Figure 4). Surprisingly, this special signature may be associated with the higher expression of ADAR2 (at least for GJC1, Figure 3D). While ADAR2 has been envisioned as a tumor suppressor, its increased expression in “relapses” would indicate that under treatment with immune checkpoint inhibitors, inhibiting ADAR2 could help prevent tumor recurrence. Some targets of ADAR2, mostly recoding editing events may be of relevance, but they could not be identified in the present study (non-significant changes in the recoding editing of COPA and COG3 and no grouping of recoding events, Supplementary Figure S2). Meanwhile, the known recoding editing that generates MHC epitopes that are recognized by anti-cancer T-cells was not downregulated, indicating the weak significance of these epitopes for the immune control of cancer. The combination of several recoding and non-coding editing events (Alu editing of GJC1) eventually mediated by ADAR2 may be responsible for the potential advantages that the overexpression of this gene provides for recurrence during treatment with immune checkpoint inhibitors. Although RNAseq data from whole tumor tissues are publicly available (for example from Liu et al. [39]), an analysis of A to G editing and its eventual correlation with clinical outcome would require the generation of tumor cell lines. Indeed, in whole tumor tissues, RNA sequences are originating not solely from tumor cells but also from non-tumor stroma cells, immune cells, etc. Thus, editing analysis of these sequencing files would not provide information exclusively on melanoma cells but would primarily reflect the heterogeneity of the tumor samples (percentages of immune cells, non-tumor cells, blood vessels, etc.) that varies from one sample to another. We foresee that our results will galvanize the analysis of A to G editing in tumor cell lines made from patients with a precisely known cancer history and will enable the identification of further correlations between editing and cancer outcome. Our study does not address the biological or biochemical phenomenon that connects the Alu-editing signature to the immunotherapeutic treatment. It however is the very first study that shows a correlation between RNA editing in melanoma and clinical outcome. We foresee that based on those observational results, further studies can be undertaken to more precisely decipher the mechanisms leading to the differential RNA editing in tumor cells during immunotherapy of cancer.

## 4. Conclusions

Our study points to differential editing in Alu sequences between melanoma cell lines obtained before therapy and melanoma cell lines made from relapses during treatment with immune checkpoint inhibitors. Those findings may be of relevance for diagnostic and prognostic tools as well as for the development of drugs or treatments that may lower the risks of relapses during therapy with immune checkpoint inhibitors.

## Figures and Tables

**Table 1:** Patients and corresponding cell lines. The table presents the patients’ demographics and treatments as well as the characteristics of the cell lines.

	Gender	Tumor	Excision Place	Cell Lines	Treatment	Immunotherapy	Duration	Cell Line Purity
Patient 1	M	Metastasis	Lymph node	MM130820	Before IT			87%
Patient 2	F	Metastasis	Trunk	MM150325	Before IT			98%
	F	Metastasis	Trunk	MM150604	Relapsed	Ipilimumab	3 weeks	74%
Patient 3	F	Primary	Lymph node	MM140905	Before IT			99%
Patient 4	M	Metastasis	Back	M130107	Before IT			59%
	M	Metastasis	Lymph node	M121102	Before IT			65%
Patient 5	F	Metastasis	Lung	M121008	Before IT			20%
Patient 6	F	Metastasis	Lymph node	MM130626	Before IT			60%
Patient 7	F	Metastasis	Leg	MM130106	Before IT			57%
	F	Metastasis	Leg	MM131205	Relapsed	Ipilimumab	3 weeks	78%
	F	Metastasis	Leg	MM131206	Relapsed	Ipilimumab	3 weeks	65%
Patient 8	M	Metastasis	Trunk	MM150405	Before IT			58%
	M	Metastasis	Lymph node	M150404	Before IT			
Patient 9	F	Metastasis	Skin	MM130926	Before IT			50%
Patient 10	F	Metastasis	Skin	M980513	Before IT			83%
Patient 11	M	Metastasis	Arm	MM140902	Relapsed	Ipilimumab	3 months	100%
	M	Metastasis	Arm	M130830	Relapsed	Ipilimumab	3 months	100%
Patient 12	M	Metastasis	Back	MM130434	Relapsed	Ipilimumab	3 months	77%
Patient 13	M	Metastasis	Trunk	MM121224	Relapsed	Ipilimumab	3 months	75%
Patient 14	M	Metastasis	Skin	M130420	Relapsed	Ipilimumab	10 days	100%
	M	Metastasis	Liver	M130421	Relapsed	Ipilimumab	10 days	100%
	M	Metastasis	Testis	M130425	Relapsed	Ipilimumab	10 days	100%
	M	Metastasis	Lung	MM130427	Relapsed	Ipilimumab	10 days	97%

**Table 2:** Demographics and characteristics of the cell lines for both groups.

Characteristic	Relapsed N = 11 ^1^	Before IT N = 12 ^1^
Age at sampling date	48 (18–60)	61 (32–86)
Sex		
F	3 (27%)	7 (58%)
M	8 (73%)	5 (42%)
Mutation		
BRAF	2 (18%)	7 (58%)
BRAF and NRAS	1 (9.1%)	0 (0%)
NRAS	8 (73%)	5 (42%)
Excision place		
Arm	2 (18%)	0 (0%)
Back	1 (9.1%)	1 (8.3%)
Leg	2 (18%)	1 (8.3%)
Liver	1 (9.1%)	0 (0%)
Lung	1 (9.1%)	1 (8.3%)
Lymph node	0 (0%)	5 (42%)
Skin	1 (9.1%)	2 (17%)
Testis	1 (9.1%)	0 (0%)
Trunk	2 (18%)	2 (17%)

^1^ Statistics presented: median (minimum-maximum); n (%).

## Data Availability

The data presented in this study are contained within the article and supplementary material.

## Supplementary Materials

The following are available online at https://www.mdpi.com/2311-553X/7/1/5/s1, Figure S1: Analysis of gene expression, Figure S2: Heat map representation of all recoding editing events, Table S1: Top 50 differentially expressed genes, Table S2: List and frequency of all editing events, Table S3: Recoding editing, Table S4: Alu editing indexes, Table S5: Top 10 differentially edited Alu, Table S6: Alu editing datasheet, Table S7: Alu editing events included in the PCA.

## Abbreviations

ADAR	adenosine deaminase acting on RNA
ADAT	adenosine deaminase acting on tRNA
Alu	Arthrobacter luteus
AZIN1	encoding antizyme inhibitor 1
CCNI	cyclin I
COG3	component of oligomeric Golgi complex 3
COPA	coatomer protein complex subunit α
FLNB	filamin B
Gabra3	gamma-aminobutyric acid type A receptor alpha 3 subunit
GJC1	Gap junction gamma-1 protein
Glur-B	glutamate receptor subunit B
HLA	human leukocyte antigen
IGFBP7	insulin-like growth factor-binding protein 7
LINE	Long interspersed element
NEIL1	DNA base excision repair glycosylase enzyme NEI-like protein 1
NSCLC	non-small cell lung carcinoma
PTPN6	protein tyrosine phosphatase non-receptor type 6
RHOQ	Ras homologue family member Q
Tils	tumor infiltrating lymphocytes

## References

[1] Hsiao Y.E., Bahn J.H., Yang Y., Lin X., Tran S., Yang E.W., Quinones-Valdez G., Xiao X. (2018). RNA editing in nascent RNA affects pre-mRNA splicing. Genome Res..

[2] Bass B.L. (2002). RNA editing by adenosine deaminases that act on RNA. Annu. Rev. Biochem..

[3] Levanon E.Y., Hallegger M., Kinar Y., Shemesh R., Djinovic-Carugo K., Rechavi G., Jantsch M.F., Eisenberg E. (2005). Evolutionarily conserved human targets of adenosine to inosine RNA editing. Nucleic Acids Res..

[4] Gerber A.P., Keller W. (2001). RNA editing by base deamination: More enzymes, more targets, new mysteries. Trends Biochem. Sci..

[5] Blanc V., Davidson N.O. (2003). C-to-U RNA editing: Mechanisms leading to genetic diversity. J. Biol. Chem..

[6] Picardi E., Manzari C., Mastropasqua F., Aiello I., D’Erchia A.M., Pesole G. (2015). Profiling RNA editing in human tissues: Towards the inosinome Atlas. Sci. Rep..

[7] Nishikura K. (2016). A-to-I editing of coding and non-coding RNAs by ADARs. Nat. Rev. Mol. Cell Biol..

[8] Athanasiadis A., Rich A., Maas S. (2004). Widespread A-to-I RNA editing of Alu-containing mRNAs in the human transcriptome. Plos Biol..

[9] Bazak L., Haviv A., Barak M., Jacob-Hirsch J., Deng P., Zhang R., Isaacs F.J., Rechavi G., Li J.B., Eisenberg E. (2014). A-to-I RNA editing occurs at over a hundred million genomic sites, located in a majority of human genes. Genome Res..

[10] Kim D.D., Kim T.T., Walsh T., Kobayashi Y., Matise T.C., Buyske S., Gabriel A. (2004). Widespread RNA editing of embedded alu elements in the human transcriptome. Genome Res..

[11] Nishikura K. (2010). Functions and regulation of RNA editing by ADAR deaminases. Annu. Rev. Biochem..

[12] Hood J.L., Emeson R.B. (2012). Editing of neurotransmitter receptor and ion channel RNAs in the nervous system. Curr. Top. Microbiol. Immunol..

[13] Levanon E.Y., Eisenberg E., Yelin R., Nemzer S., Hallegger M., Shemesh R., Fligelman Z.Y., Shoshan A., Pollock S.R., Sztybel D. (2004). Systematic identification of abundant A-to-I editing sites in the human transcriptome. Nat. Biotechnol..

[14] Wang Q., Khillan J., Gadue P., Nishikura K. (2000). Requirement of the RNA editing deaminase ADAR1 gene for embryonic erythropoiesis. Science.

[15] Higuchi M., Maas S., Single F.N., Hartner J., Rozov A., Burnashev N., Feldmeyer D., Sprengel R., Seeburg P.H. (2000). Point mutation in an AMPA receptor gene rescues lethality in mice deficient in the RNA-editing enzyme ADAR. Nature.

[16] Wahlstedt H., Daniel C., Enstero M., Ohman M. (2009). Large-scale mRNA sequencing determines global regulation of RNA editing during brain development. Genome Res..

[17] Roth S.H., Danan-Gotthold M., Ben-Izhak M., Rechavi G., Cohen C.J., Louzoun Y., Levanon E.Y. (2018). Increased RNA Editing May Provide a Source for Autoantigens in Systemic Lupus Erythematosus. Cell Rep..

[18] Tusup M., Kundig T., Pascolo S. (2018). Epitranscriptomics of cancer. World J. Clin. Oncol..

[19] Maas S., Kawahara Y., Tamburro K.M., Nishikura K. (2006). A-to-I RNA editing and human disease. RNA Biol..

[20] Keegan L.P., Gallo A., O’Connell M.A. (2001). The many roles of an RNA editor. Nat. Rev. Genet..

[21] Han L., Diao L., Yu S., Xu X., Li J., Zhang R., Yang Y., Werner H.M.J., Eterovic A.K., Yuan Y. (2015). The Genomic Landscape and Clinical Relevance of A-to-I RNA Editing in Human Cancers. Cancer Cell.

[22] Paz-Yaacov N., Bazak L., Buchumenski I., Porath H.T., Danan-Gotthold M., Knisbacher B.A., Eisenberg E., Levanon E.Y. (2015). Elevated RNA Editing Activity Is a Major Contributor to Transcriptomic Diversity in Tumors. Cell Rep..

[23] Mannion N.M., Greenwood S.M., Young R., Cox S., Brindle J., Read D., Nellåker C., Vesely C., Ponting C.P., McLaughlin P.J. (2014). The RNA-editing enzyme ADAR1 controls innate immune responses to RNA. Cell Rep..

[24] Ishizuka J.J., Manguso R.T., Cheruiyot C.K., Bi K., Panda A., Iracheta-Vellve A., Miller B.C., Du P.P., Yates K.B., Dubrot J. (2019). Loss of ADAR1 in tumours overcomes resistance to immune checkpoint blockade. Nat. Cell Biol..

[25] Liu H., Golji J., Brodeur L.K., Chung F.S., Chen J.T., Debeaumont R.S., Bullock C.P., Jones M.D., Kerr G., Li L. (2019). Tumor-derived IFN triggers chronic pathway agonism and sensitivity to ADAR loss. Nat. Med..

[26] Zhang M., Fritsche J., Roszik J., Williams L.J., Peng X., Chiu Y., Tsou C.-C., Hoffgaard F., Goldfinger V., Schoor O. (2018). RNA editing derived epitopes function as cancer antigens to elicit immune responses. Nat. Commun..

[27] Ribas A., Wolchok J.D. (2018). Cancer immunotherapy using checkpoint blockade. Science.

[28] Raaijmakers M.I.G., Widmer D.S., Maudrich M., Koch T., Langer A., Flace A., Schnyder C., Dummer R., Levesque M.P. (2015). A new live-cell biobank workflow efficiently recovers heterogeneous melanoma cells from native biopsies. Exp. Dermatol..

[29] Dobin A., Davis C.A., Schlesinger F., Drenkow J., Zaleski C., Jha S., Batut P., Chaisson M., Gingeras T.R. (2013). STAR: Ultrafast universal RNA-seq aligner. Bioinformatics.

[30] Law C.W., Chen Y., Shi W., Smyth G.K. (2014). voom: Precision weights unlock linear model analysis tools for RNA-seq read counts. Genome Biol..

[31] Picardi E., Pesole G. (2013). REDItools: High-throughput RNA editing detection made easy. Bioinformatics.

[32] D’Antonio M., Picardi E., Castrignanò T., D’Erchia A.M., Pesole G. (2014). Exploring the RNA Editing Potential of RNA-Seq Data by ExpEdit. Adv. Struct. Saf. Stud..

[33] Picardi E., D’Antonio M., Carrabino D., Castrignanò T., Pesole G. (2011). ExpEdit: A webserver to explore human RNA editing in RNA-Seq experiments. Bioinformatics.

[34] Zaranek A.W., Levanon E.Y., Zecharia T., Clegg T., Church G.M. (2010). A Survey of Genomic Traces Reveals a Common Sequencing Error, RNA Editing, and DNA Editing. PLoS Genet..

[35] Shelton P.M., Duran A., Nakanishi Y., Reina-Campos M., Kasashima H., Llado V., Ma L., Campos A., Garcia-Olmo D., Garcia-Arranz M. (2018). The Secretion of miR-200s by a PKCzeta/ADAR2 Signaling Axis Promotes Liver Metastasis in Colorectal Cancer. Cell Rep..

[36] Galeano F., Rossetti C., Tomaselli S., Cifaldi L., Lezzerini M., Pezzullo M., Boldrini R., Massimi L., Di Rocco C.M., Locatelli F. (2013). ADAR2-editing activity inhibits glioblastoma growth through the modulation of the CDC14B/Skp2/p21/p27 axis. Oncogene.

[37] Peng X., Xu X., Wang Y., Hawke D., Yu S., Han L., Zhou Z., Mojumdar K., Jeong K.J., Labrie M. (2018). A-to-I RNA Editing Contributes to Proteomic Diversity in Cancer. Cancer Cell.

[38] Anantharaman A., Tripathi V., Khan A., Yoon J.-H., Singh D.K., Gholamalamdari O., Guang S., Ohlson J., Wahlstedt H., Öhman M. (2017). ADAR2 regulates RNA stability by modifying access of decay-promoting RNA-binding proteins. Nucleic Acids Res..

[39] Liu D., Schilling B., Liu D., Sucker A., Livingstone E., Jerby-Arnon L., Zimmer L., Gutzmer R., Satzger I., Loquai C. (2019). Integrative molecular and clinical modeling of clinical outcomes to PD1 blockade in patients with metastatic melanoma. Nat. Med..

