# Correction: Tusup et al. Evaluation of the Interplay between the ADAR Editome and Immunotherapy in Melanoma. *Non-Coding RNA* 2021, *7*, 5

**DOI:** 10.3390/ncrna8060079

**Published:** 2022-11-21

**Authors:** Marina Tusup, Phil F. Cheng, Ernesto Picardi, Austeja Raziunaite, Reinhard Dummer, Mitchell P. Levesque, Lars E. French, Emmanuella Guenova, Thomas M. Kundig, Steve Pascolo

**Affiliations:** 1 Department of Dermatology, University Hospital of Zürich, Gloriastrasse 31, 8091 Zürich, Switzerland; 2 Faculty of Medicine, University of Zürich, 8091 Zürich, Switzerland; 3 Department of Biosciences, Biotechnology and Biopharmaceutics, University of Bari “A. Moro”, 70121 Bari, Italy; 4 Institute of Biomembranes, Bioenergetics and Molecular Biotechnologies (IBIOM), National Research Council, 70126 Bari, Italy; 5 Department of Dermatology and Allergy, University Hospital, LMU Munich, 80336 Munich, Germany; 6 Department of Dermatology, Lausanne University Hospital and Faculty of Biology and Medicine, University of Lausanne, 1015 Lausanne, Switzerland

In the original article [1], there were two mistakes in the published version of Table 1. For patient 3, the tumor should be “Metastasis” instead of “Primary”; for patient 10, the excision place should be “Lymph Node” instead of “Skin”. The corrected Table 1 appears below. 

**Table 1:** Patients and corresponding cell lines. The table presents the patients’ demographics and treatments as well as the characteristics of the cell lines.

	Gender	Tumor	Excision Place	Cell Lines	Treatment	Immunotherapy	Duration	Cell Line Purity
Patient 1	M	Metastasis	Lymph node	MM130820	Before IT			87%
Patient 2	F	Metastasis	Trunk	MM150325	Before IT			98%
	F	Metastasis	Trunk	MM150604	Relapsed	Ipilimumab	3 weeks	74%
Patient 3	F	Metastasis	Lymph node	MM140905	Before IT			99%
Patient 4	M	Metastasis	Back	M130107	Before IT			59%
	M	Metastasis	Lymph node	M121102	Before IT			65%
Patient 5	F	Metastasis	Lung	M121008	Before IT			20%
Patient 6	F	Metastasis	Lymph node	MM130626	Before IT			60%
Patient 7	F	Metastasis	Leg	MM130106	Before IT			57%
	F	Metastasis	Leg	MM131205	Relapsed	Ipilimumab	3 weeks	78%
	F	Metastasis	Leg	MM131206	Relapsed	Ipilimumab	3 weeks	65%
Patient 8	M	Metastasis	Trunk	MM150405	Before IT			58%
	M	Metastasis	Lymph node	M150404	Before IT			
Patient 9	F	Metastasis	Skin	MM130926	Before IT			50%
Patient 10	F	Metastasis	Lymph node	M980513	Before IT			83%
Patient 11	M	Metastasis	Arm	MM140902	Relapsed	Ipilimumab	3 months	100%
	M	Metastasis	Arm	M130830	Relapsed	Ipilimumab	3 months	100%
Patient 12	M	Metastasis	Back	MM130434	Relapsed	Ipilimumab	3 months	77%
Patient 13	M	Metastasis	Trunk	MM121224	Relapsed	Ipilimumab	3 months	75%
Patient 14	M	Metastasis	Skin	M130420	Relapsed	Ipilimumab	10 days	100%
	M	Metastasis	Liver	M130421	Relapsed	Ipilimumab	10 days	100%
	M	Metastasis	Testis	M130425	Relapsed	Ipilimumab	10 days	100%
	M	Metastasis	Lung	MM130427	Relapsed	Ipilimumab	10 days	97%

The authors apologize for any inconvenience caused and state that the scientific conclusions are unaffected. This correction was approved by the Academic Editor. The original publication has also been updated.
