# Supplementary material for: Evaluation of the Interplay between the ADAR Editome and Immunotherapy in Melanoma
Source: Noncoding RNA. 2021 Jan 12;7(1):5. doi: 10.3390/ncrna7010005 (PMC7838980; doi:10.3390/ncrna7010005)
Supplement: Supplementary file 1 [file ncrna-07-00005-s001.zip › Supplementary Figure1 and Figure2.pdf]

non-coding RNA

MDPI

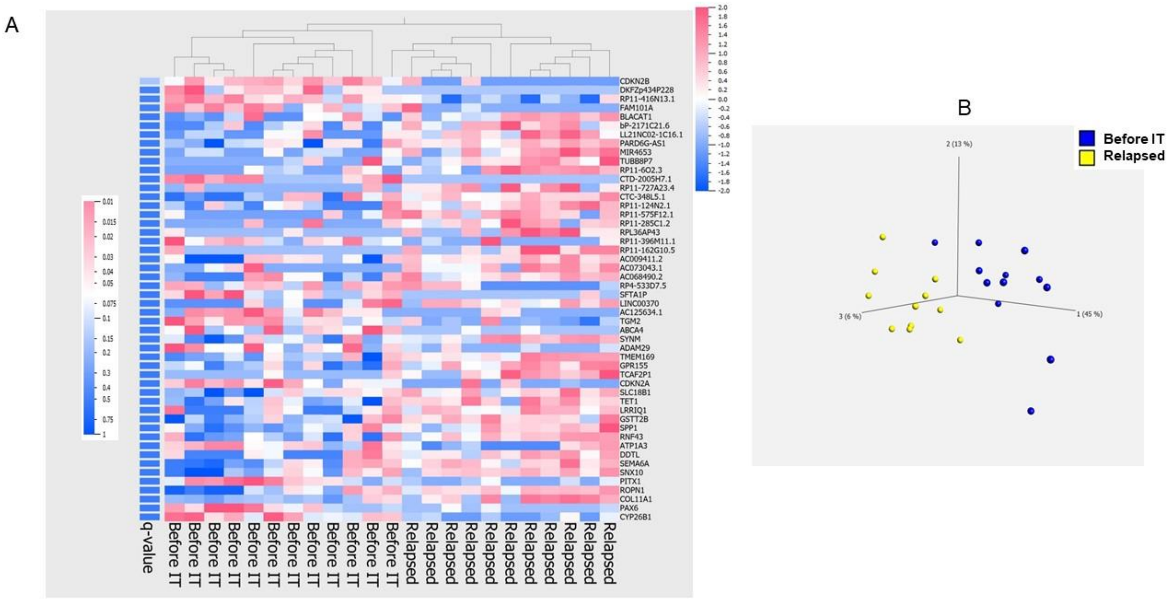

Supplementary Figure S1. Analysis of gene expression. The graph in A symbolizes the level of gene expression for the 50 genes most differentially expressed between cell lines made from tumours before IT and cell lines made from tumours relapsing during immunotherapy ("relapsed"). Cell lines are organized via gene expression pattern hierarchical clustering. Genes are ranked based on q values obtained through multivariant analysis using limma and voom test. The scale on the left represents the q-value. The scale on the right represents the log2 scale of gene expression where red denotes high expression and blue denotes low expression. In B a 3D PCA plot representation of samples based on top 50 genes differentially expressed.

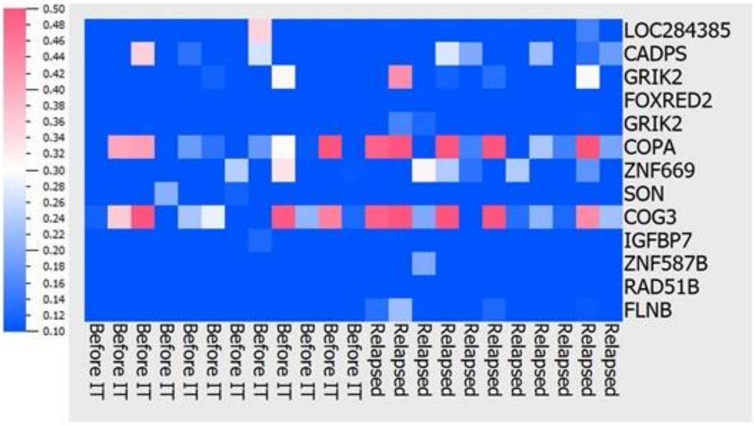

Supplementary Figure S2. Heat map representation of all recoding editing events. Thirteen editing recoding events were evidenced in cell lines made from tumours before immunotherapy ("Before IT") and from relapses occurring during immunotherapy ("Relapsed"). Editing is observed in the majority of cell lines for only COG3 and COPA. The scale on the left indicates the level of editing observed
per site.
